# Qualitative service evaluation of a multimodal pilot service for early detection of liver disease in high-risk groups: ‘Alright My Liver?’

**DOI:** 10.1136/bmjgast-2024-001560

**Published:** 2024-11-12

**Authors:** Ann Jane Archer, Tom May, Hannah Bowers, Joanna Kesten, Sally Tilden, Kushala Abeysekera, Fiona H Gordon, Matthew Hickman, Lucy Yardley

**Affiliations:** 1 Bristol Medical School, University of Bristol, Bristol, UK; 2 Department of Liver Medicine, Bristol Royal Infirmary, Bristol, UK; 3 NIHR Health Protection Research Unit in Behavioural Science and Evaluation, University of Bristol, Bristol, UK; 4 Department of Liver Medicine, University Hospitals Bristol and Weston NHS Foundation Trust, Bristol, UK

**Keywords:** LIVER, SCREENING, ALCOHOLIC LIVER DISEASE, Non-alcoholic Fatty Liver Disease, LIVER CIRRHOSIS

## Abstract

**Objective:**

Liver disease is a growing cause of premature death in the UK. The National Health Service in England (NHS England) has funded regional early detection programmes through Community Liver Health Check pilots. ‘Alright My Liver?’ is Bristol and Severn’s pilot service offering early detection of liver disease through screening events serving populations at risk, including people with a history of drug or alcohol use, type 2 diabetes and obesity. The service offers point-of-care testing for liver disease and a supported follow-up process.

**Methods:**

Semistructured interviews were conducted with 14 service users and six service providers over a 6-month period using diversity sampling. Topic guides encouraged discussion of experiences of the service as well as barriers and facilitators to accessing the service. Data were analysed using thematic analysis, and positive and negative comments pertaining to the service were collated in a ‘table of changes’ to inform optimisation.

**Results:**

Three main themes were identified: (1) motivations for engagement, (2) experience of the service and (3) health impacts. Key motivations for engagement were screening as a novel opportunity, a response to immediate health concerns or as reassurance. Service users commented on its convenience and that staff interactions were warm and informative. Some felt that follow-up could be more intensive. Impacts varied depending on perceived risk factors and screening results but generally involved stating a commitment to healthy lifestyle changes, including reducing alcohol use.

**Conclusion:**

Targeted screening for liver disease in high-risk groups through this pilot service was deemed an appropriate and accessible intervention, with important optimisations identified.

**WHAT IS ALREADY KNOWN ON THIS TOPIC**

**WHAT THIS STUDY ADDS**

- This study reports on the factors motivating people with risk factors for liver disease to engage with screening and their suggestions for service optimisations.
- The study findings suggest that some service users felt motivated to adopt healthier lifestyles following screening, regardless of the result.

**HOW THIS STUDY MIGHT AFFECT RESEARCH, PRACTICE OR POLICY**

- The findings suggest that community-based screening for liver disease in at-risk populations is welcomed and may have positive health impacts.

## Introduction

 Chronic liver disease is now a leading cause of death of British 35–49 year olds, overtaking suicide.^1^ It is a disabling illness with a major symptom burden for those affected^2^ and has a significant impact on health services and the economy worldwide. In 2017 alone, chronic liver disease accounted for 41.4 million disability-adjusted life years compared with 35.8 million for chronic kidney disease.^3^The majority of people with chronic liver disease are first diagnosed when they are admitted to a hospital with an emergency presentation of a liver-related complication,^4^ at which point mortality is one in six, much higher than in the long asymptomatic phase.^5^ Cirrhosis prevalence is <1% in the general population, implying that a targeted approach to case finding is needed.^6 7^

Common risk factors for chronic liver disease include obesity, type 2 diabetes, harmful alcohol use and viral hepatitis. Viral hepatitis includes hepatitis B (HBV), which is most prevalent among migrant populations^8^ and over 85% of hepatitis C in the UK is in people with a history of injecting drug use.^9^ There is a strong association between many of these risk factors and deprivation, and people experiencing deprivation have been found to have a four-fold increased mortality risk in chronic liver disease in the UK.^10^ It is therefore essential that early detection programmes are acceptable^11^ and accessible to people in underserved and deprived communities, as well as those with specific risk factors.^12^

Early diagnosis of liver disease can improve outcomes. For alcohol-related liver disease (ARLD), abstinence from alcohol is essential to halting disease progression, which can often be prompted by a diagnosis of cirrhosis.^13^ In metabolic dysfunction-associated liver disease (MASLD), weight loss and optimisation of diabetic control and lipids can slow disease progression.^14^ In MASLD with increased alcohol intake (MetALD), all of these risk factors must be addressed, which is pertinent in a population who may not view themselves as using alcohol excessively. Successful treatment of hepatitis C can reverse fibrosis and cirrhosis, while controlling disease activity and preventing complications like hepatocellular carcinoma (HCC) is the key to managing HBV.^15 16^ People with cirrhosis can benefit from regular outpatient appointments for advice and education, assessment for transplant suitability and close monitoring for complications including surveillance for varices and HCC.^17^

There is evidence that transient elastography (TE) is an acceptable liver screening measure in the primary care setting^18^ and as part of hepatitis C testing and treatment.^19^ Screening for liver disease in the primary care setting has been shown to be acceptable, and the role of nurse-led intervention and peer support is well-established in hepatitis C testing and treatment,^20 21^ but outreach-based screening has not been studied in an all-cause cirrhosis detection context.

The ‘Alright My Liver?’ pilot is a novel approach to early detection, encompassing liver disease of all aetiologies in people from underserved communities across multiple settings. This paper presents findings from a service evaluation exploring service users’ and service providers’ views and experiences of the service and presents suggested optimisations for wider implementation of the service.

## Methods

### Setting

In 2022, NHS England funded the Bristol and Severn hepatitis C Operational Delivery Network (ODN) to broaden its existing outreach work as part of the ‘Piloting Community Liver Health Checks’ programme at 12 sites nationally.^22^ The Bristol and Severn ODN developed the regional ‘Alright My Liver?’ service in collaboration with service users to screen for chronic liver disease in populations at risk.

Liver health screening events were co-located with existing services that support underserved and high-risk groups. These included drug and alcohol services, primary care services in areas with a high index of deprivation and a community health organisation primarily providing health outreach to black and ethnic minority communities in the region. Events were promoted through posters on local bus services and in community services, through general practice (GP) text tools and on a dedicated website.^23^ Word-of-mouth promotion particularly through community champions and support works was also encouraged.

An ‘Alright My Liver?’ outreach assessment comprises a health history, TE using FibroScan and capillary blood-borne virus testing if indicated. These assessments take place in a clinic room, in a private ambulance or in a screened area (eg, if the screening event is at a community centre).

TE is a point-of-care non-invasive test of liver stiffness as a surrogate of fibrosis. It has excellent receiver operator characteristics for the diagnosis of steatotic liver disease and viral hepatitis, the most common causes of chronic liver disease.^24^,^26^ It takes <5 min to perform and is similar in experience to an ultrasound scan. An elevated liver stiffness measurement by TE can be used to diagnose chronic liver disease when combined with other tests including full clinical history and examination. All patients received personalised advice including brief alcohol reduction interventions^27 28^ and local drug and alcohol service signposting if indicated.

**Figure 1:**
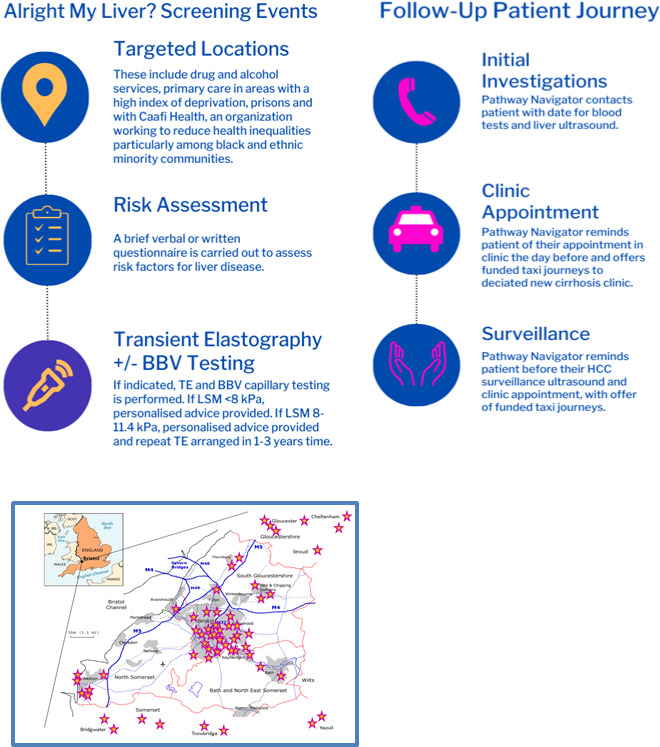
The ‘Alright My Liver?’ care pathway. HCC, hepatocellular carcinoma; BBV, blood borne viruses; TE, transient elastography; LSM, liver stiffness measurement.

Service users found to have an abnormal TE result are booked directly into the hepatology clinic and provided with telephone reminders through a dedicated pathway navigator, who would also communicate with service users’ key workers or relatives with their consent. The pathway navigator was also able to offer funded transport to and from appointments (see figure 1). For those confirmed to have liver disease, lifelong follow-up is implemented.

### Service evaluation design

The service evaluation employed a qualitative design using semistructured remote telephone/ video call methods with 14 service users and six service providers. Interviews with both groups were carried out between December 2022 and May 2023, and explored experiences of the service, including barriers and facilitators to uptake and engagement and any perceived health impacts and benefits. The service evaluation used elements of the Agile Co-production and Evaluation (ACE) Framework, a novel approach to rapidly developing inclusive public health interventions, messaging and guidance. Specific ACE methods are published elsewhere,^29^ but elements used in this work are outlined below.

The single-site service evaluation was registered with the University Hospitals Bristol and Weston NHS Trust (GASHEP/SE/2022-23/03) and did not require Health Research Authority approvals.

### Patient and public involvement

Two public involvement groups were established and included three people identified through a community drug and alcohol service, and two members of the general public recruited via social media. Of the five contributors, three identified as male and two as female. The majority were of Black African/Caribbean heritage (n=4), one was white British.

For each group, a single, in-person meeting was held in a community centre location, which began with an ‘Alright My Liver?’ staff member providing an overview of the service in its earliest form. The groups were then invited to discuss and provide feedback on the service, and views on service evaluation materials were also obtained. Both groups were facilitated by a member of the research team (TM).

Unlike the qualitative interviews, these meetings did not seek to achieve detailed feedback on all aspects of the intervention or refine the interventions. Instead, contributors were invited to generate ideas and raise any concerns about the service and its procedures, including its delivery, location and advertising. These insights were then collated into a modified intervention planning table and fed back and discussed with ‘Alright My Liver?’ staff to help inform decisions about the service design and procedures. The nature of the ‘Alright My Liver?’ as a new pilot service meant that many of these insights were implemented as the pilot evolved. Some of these improvements happened during the study period, meaning that they are reflected in the subsequent qualitative data collected.

The insights also informed the qualitative interview topic guide by identifying key areas where additional insights from service users and staff would be beneficial, thereby enabling perceptions of intervention content to be explored in more depth and inform further optimisations (described below).

### Sample and recruitment

Service user eligibility was based on whether the person had either (1) been offered FibroScan at a public or targeted ‘Alright My Liver?’ event (2) aged 18 years of age and (3) living in the UK. For service providers, eligibility was based on (1) whether they worked directly with or closely in the delivery of the ‘Alright My Liver?’ service (2) aged 18 years or over and (3) living in the UK. In both groups, a diverse sample was sought to increase the breadth of views obtained, for instance, people with abnormal TE results.

The service evaluation used multiple recruitment methods to ensure a diverse sample in terms of age, gender, ethnicity and health status. This included opportunistic interviewing methods, whereby TM and JK attended a range of ‘Alright My Liver?’ events and approached eligible participants for the interview. Efforts were made to include potential participants who had declined the intervention, whereby an interviewer would approach these individuals immediately after they declined and ask if they would consider taking part in a qualitative interview. ‘Alright My Liver?’ staff also informed service users about the evaluation and opportunity to participate in an interview, either on-site or at a later, prearranged time via phone/remote methods. If the latter, contact details for the service evaluation team were shared if the participant suggested that they were interested in participating.

Similar methods were used for the recruitment of staff, whereby ‘Alright My Liver?’ staff advertised the service evaluation via email to staff mailing lists. A £10 voucher was offered to participants as thanks for their time.

Participants were provided with both verbal and written information about the purpose of the service evaluation, and informed that their involvement was voluntary. All participants signed a consent form to indicate their agreement to participate and provided demographic information.

Concurrent analysis alongside data collection helped the team determine the adequacy of the sample size. The principles of information power were used to support the decision to end data collection. Information power suggests that the more information in the data relating to the focus of the work, the smaller the sample size needs to be.^30^ This decision was also informed by pragmatic considerations of resources and timescales.

### Data collection and interview procedures

Interviews were conducted by TM (research fellow) and JK (research fellow) in person or via telephone or video call. TM and JK are experienced qualitative researchers educated to PhD level, with experience in drug and alcohol research and research with marginalised/under-represented groups. Interviews followed a flexible topic guide (see onlinesupplemental materials 1  2) with open-ended questions to encourage discussion of experiences of the service as well as barriers and facilitators to accessing the service. Questions were used to prompt suggestions for the optimisation of the service (eg, ‘what do you think of the location of this service?’). The decision to end data collection was informed by considerations of the sample diversity, range of views captured in the data and pragmatic considerations of resources.

### Data analysis

Interviews were recorded and transcribed verbatim. The data were analysed using a thematic approach.^31^ Transcripts were read and re-read by a qualitative researcher (HB) who conducted line-by-line coding (ie, attributing labels to units of meaning) in NVivo software. A portion of transcripts were read by TM who developed overarching themes from the coding frame through discussion with HB, JK and AA.

Positive and negative comments about the service and potential optimisations were tabulated in a table of changes (see online supplemental material 3).^32^ Data from qualitative participants were considered alongside earlier feedback from the patient and public involvement (PPI) groups. Comments were discussed with the team and changes to the service were agreed and allocated a level of priority using MoSCoW rating (Must, Should, Could or Would Have).

## Results

### Participants

20 interviews were conducted with both service users (n=14) and service providers (n=6). Interviews were conducted across a range of locations, including mobile van and community outreach events (n=8), in-patient liver unit,^2^ drug and alcohol settings^3^ and a hostel. One participant was signposted to screening at a community event by their GP. Half the service users were white British (n=7) and the majority either self-identified as current or previous users of alcohol (n=10). Five participants reported MASLD risk factors. Service providers were from various occupational backgrounds, including clinical, management and frontline staff. Tables1  2 provide overviews of participant characteristics.

**Table 1:** Characteristics of service users

Characteristics		Range/n (mean)
Sex	Female	4
	Male	10
Age	Median	48.2 (range 32–65)
Ethnicity	white British	7
	black African	4
	black Caribbean	1
	Indian	1
	Irish	1
Self-reported MASLD risk associations*	Diabetes	4
	Obesity/overweight	3
	Heart disease	2
	Sleep apnoea	1
	High cholesterol	1
	Chronic obstructive pulmonary disease	1
	Asthma	1
Employment status	Unable to work due to disability/illness	5
	Unemployed and seeking work	3
	Retired	2
	Self-employed	1
	Part time employed	2
	Full time employed	1
Education level	After finishing university	1
	After finishing college/sixth form or equivalent	6
	After finishing school	5
	Before finishing school	1
	Not known	1
Self-reported problematic substance-use history	Alcohol (including current and previous)	10
	Heroin/crack	2
Screening outcome	Abnormal result	6
	Normal result	8

* Some service users reported multiple MASLD risk associations.

MASLDmetabolic dysfunction-associated liver disease

**Table 2:** Occupation of service providers

Involvement in pilot	N
Specialist nurse	1
Pathway navigator	1
Hepatologist	1
Healthcare provider for drug and alcohol service	1
GP with specialism in inclusion health	1
Managing director local healthcare service	1

GPgeneral practice

### PPI input

A key issue identified was around service uptake, particularly for those with low health literacy about liver disease. Some concern was expressed that the service could be perceived as promoting abstinence and trying to reduce alcohol, which could affect uptake among those who drank alcohol heavily and were unwilling to stop or were fearful of an abnormal result. Strategies to overcome these issues included improved access to information that was clear and used non-technical language, as well as a need to emphasise the health benefits of liver screening without focusing on alcohol consumption. Other issues, mainly relating to the accessibility of the service (eg, in terms of locations and times) and its promotion (eg, visibility and awareness of materials) were discussed and used to inform potential adaptations to improve access (eg, rotating locations and flexible appointments) and increasing visibility of promotional materials (eg, multilingual materials in high-traffic areas, engaging local influencers).

### Thematic analysis

The thematic analysis identified three main themes, which follow the patient pathway through ‘Alright My Liver?’ (1) motivations for engagement, (2) experience of service and (3) health impacts. These are presented below.

#### Motivations for engagement

Many service users, who had self-reported histories of drug and alcohol use, expressed interest in understanding and learning about possible liver damage caused by current or previous hazardous drinking. While most of this group were asymptomatic, the majority understood the link between alcohol use and chronic liver disease, which influenced their decision to undergo screening:

In the back of my mind I thought I’d better check my liver out because I’m 54 now. I’ve drunk enough to have caused damage…I was coming back from the cinema on the bus one night, and I saw ‘Alright My Liver?’, and I thought, I’ll have a look at that (male, white British, normal result, alcohol use).

At times, some were fearful of receiving a test due to concerns that they may have chronic or severe liver damage. However, healthcare practitioners helped prompt uptake, emphasising that early intervention and management could slow down or halt the progression of cirrhosis and improve prognosis:

There’s definitely the homeless population, people hated going to hospital in general, but I think with ‘Alright My Liver?’ they get welcomed don’t they…and what we’re aiming is for this to pick up something at a stage where something can be done, and I think that they feel differently about that (GP, female).

Other service users were motivated to use the service in response to immediate health concerns or symptoms. Generally, this group presented low awareness of liver damage/disease and uptake was prompted only after the presentation of liver-related complications or symptoms. One of these participants was signposted to the service by their GP (*I was referred from my GP because I had been drinking for a while and they wanted to make sure that my liver was OK,* male, white British, normal result, MASLD risk, alcohol use), while others identified the service as a convenient alternative to primary care screening:

It’s more quicker and it gives you the results straight away. I think I’m not going to try to say better than a GP but the GP sometimes takes ages and make you like really depressed and stressed and you’re worried about the result and stuff but this one like immediately you know about yourself (male, black African, normal result, alcohol use).

Finally, due to some screening events occurring at community health clinics, a small number of service users chose to undergo a liver screen alongside other routine health checks offered at clinics, regardless of any doubt or anxiety related to the health of their liver. Among this group, uptake was largely opportunistic and facilitated by the novelty, accessibility and convenience of the intervention (see ‘a convenient opportunity’). There was a perception among these participants that had they not attended a community health clinic event, they would not have undergone or sought a screen elsewhere (eg, at a GP). Health promotion practitioners working at events were also influential in prompting uptake:

I was thinking all day perhaps I ought to get it done. I had my test for diabetes and my blood pressure and they said, ooh, do you want to go over and have your liver screened.It was just like by chance really. So I thought it was a good idea while I’m here perhaps if I could get it done (female, white British, abnormal result, suspected MASLD).

#### Experience of service

This theme describes aspects of the service that service users identified as positive and negative in their experience. This theme includes three sub-themes: ‘A convenient opportunity’, ‘Provision of information’ and ‘Interpersonal engagement’.

##### A convenient opportunity

Service users described the service as quick with a rapid result (*it’s free and available and convenient*, *Male, White British, MASLD risk, Alcohol use, Normal result*), and provided an opportunity to learn more about one’s health that might not be provided elsewhere. Some service users also referenced long wait times for appointments through primary care or hospital services, whereas this opportunity was immediately available:

R: Have you ever tried to get your liver scanned anywhere else, at the doctors, at the hospital or anywhere like that?

P: Not to be honest because sometimes appointment is taking ages like a couple of months and stuff but this one is like really easy, easy and quick (male, black African, normal result, MASLD risk, alcohol use).

Service users commented on how it was convenient for them to engage with the service when it was located nearby or within another service they were using (eg, with drug and alcohol services). They suggested that working in these locations would improve engagement as people who might be less likely to engage with healthcare services generally will be more likely to engage if the service is located somewhere very convenient for them to attend:

Yes, because there’s a lot of people that are in here, I imagine a lot of them have got to be here because through probation or through a court order, you know, and if there’s an opportunity because a lot of them are druggies and they don't really want to get clean and they're not bothered about going to the doctors or hospitals or things like that and with you guys that’s come today, they've got that opportunity (male, white British, normal result, alcohol+heroin/crack use).

One service provider described how bringing the service to a variety of settings was beneficial in raising awareness of liver disease and the service:

I think as well, the events we’ve done with [Community Organisation], they’ve been fantastic in reaching a really diverse set of communities and amazing at raising awareness, particularly some of the events that we go to where it may be higher populations of people that are Muslims, who actually don’t tend to drink alcohol, still really brilliant that we can spread the message. It’s not just alcohol. There’s lots of other risk factors that could lead to cirrhosis (specialist nurse, female).

Many service users had useful suggestions for potential future locations such as supermarket car parks, which they felt would be facilitated by the use of the mobile testing unit. There was a feeling that these locations would increase uptake of the service:

That’s a very good spot for this because I go to do shopping from Asda and I know a lot of people there and for definite they’re going to come check their liver (male, black African, normal result, MASLD risk, alcohol use).

For the follow-up appointments for service users with abnormal results, there was a sense that the delay between the scan and the follow-up appointment was too long:

She did say I was gonna be on medication, which I’ve not heard anything and also, I’d be engaged with someone to talk to me about it, but I’ve not heard anything about that either (female, white British, abnormal result, suspected MASLD).

A service provider reported that this negative experience was mitigated by phone calls between appointments, and one service user commented that these provided continuity:

[Service Provider Name] was the one who had organised the appointments in the beginning, and we’d been on the phone. I wasn’t actually expecting her to be there. It was like quite nice (male, Irish, abnormal result, suspected ARLD).

The ones that we have, we, sort of, identified at the beginning, like when I first started, I built up a good relationship with them, they know when I’m phoning them, they know it’s me on the phone now, and almost having a bit of a chit-chat about what’s going on and stuff (liver surveillance support worker, female).

One participant also commented that the offer of a taxi to attend their follow-up was crucial and they would not have attended without it:

No I don’t think I probably would have [attended if not for taxi provision]. Yes the charge, I mean I’d have to park up on top of some (place name) but it made it all very convenient for me to go (male, white British, abnormal result, suspected MetALD).

##### Provision of information

For service users with a normal result, many felt that the level of information they were given prior to the scan was appropriate:

She explained it really well and told me what it would feel like and not to worry and she was very good, yeah, she’s absolutely brilliant (male, white British, normal result, alcohol+heroin/crack use).

There were mixed views on the use of a leaflet, with some appreciating and some not engaging with written information:

I think they’re all helpful because usually at the back of them all I think they have the websites and other affiliations and other things that you can look into if you want. So yeah, the leaflets are good (male, Irish, abnormal result, suspected ARLD).

No, they asked me if I wanted some leaflets to read and I said, I’m not really interested. I don’t really read them. (male, white British, abnormal result, suspected ARLD).

For participants with an abnormal result, there was a sense that they wanted more information during the initial scan, particularly because there was a long wait for a follow-up appointment. They reported making changes to their lifestyle based on their own assumptions as opposed to any advice they had been given. One participant in particular seemed unclear about his result and what it meant:

Yes I don’t know whether I’ve got to give up drinking forever or am I doing it wrong like when I go on holiday and having a drink and that, am I harming myself (male, white British, abnormal result, suspected MetALD).

##### Interpersonal engagement

Service users highlighted how important outreach via community services was to their engagement with their screening. However, this did result in some locations lacking adequate privacy for service users:

Like some people just don’t want other people knowing their stuff and it seems quite open. It seems a bit open in here. People are walking through. There’s no privacy in there (male, white British, abnormal result, suspected ARLD).

Word-of-mouth within communities was seen as a useful strategy for engaging with people:

Yeah, yeah that is good. Everybody at the café, restaurant café I tell everybody you have a scan on my liver (male, black African, normal result).

A trusting relationship was seen as an important facilitator to engagement. In particular, service users talked about the friendly nature of service providers that helped them feel at ease:

She knows how to approach people and you know influence them and all that, that’s how she got me the first time, everything was very well and bingo finally I’m like all my people you have to do this thing you and I’m going to bring you all, I’m gonna bring you all my people (male, black African, normal result).

Collaboration with existing community services was reported to help the service be appropriate and accessible by prospective clients:

Yes, we have a relatively big clinic room, they set up down there. And we shuffle people towards them when they come in. So that’s been quite nice because I think lots of our clients sort of see it as part of the service as well rather than an external agency. Which I think also helps bring a bit of trust in as well which works quite well (healthcare provider, drug and alcohol service, female).

### Perceived health and social impacts

A range of potential health impacts was cited in response to screening results. Among service users with no MASLD risk factors or alcohol/drug use, those with normal results described the result as expected but reported feeling reassured that no significant health concerns had been identified:

when I checked I was worried at the beginning about what they were going to say, what was going on. So, you know when you go for a check-up and you’re getting a bit panicky. When I was coming out I was really relieved, when they checked it yeah I was relieved (female, black African, normal result).

Similarly, service users who recognised that they could be at risk of liver damage prior to the screen (due to the presence of MASLD risk factors, engagement in risk behaviours or signposting) but received a normal result expressed feelings of relief that no significant damage to their liver had been identified:

That’s like a phew, and it’s like a relief …I can go to bed now and know there’s nothing wrong with my liver and it’s a good thing, it really is a good thing (male, white British, normal result, alcohol+heroin/crack use).

For this group, some described how a normal result prompted the consideration of health and lifestyle changes to reduce the risk of any future liver damage:

I’m going to stop alcohol for good, that’s number one. Number two I’m so happy and relieved and number three I’m not gonna damage anything from my inside or something is really going to affect me I’m not gonna do it. (male, black African, normal result, MASLD risk, alcohol use).

Responses to abnormal results also varied depending on the perceived level of risk among service users prior to screening. Among those who perceived themselves as low risk, many expressed surprise when notified of an abnormal result, as they did not consider themselves to have risk factors for either MASLD or ARLD. For instance, one service user, who stated that they consumed 'two tins a night', reported:

[feeling] a bit cheated to be quite honest that I’m not a big drinker yet I’ve got cirrhosis of the liver (male, white British, abnormal result, suspected MetALD).

Nevertheless, these service users were thankful that a diagnosis had been made so that future lifestyle changes could be adopted:

It sort of made me realise that I have gotta go a bit more careful with what I eat and do…I’ve taken swimming early morning. Doing 22 lengths at 5:30am (female, white British, abnormal result, suspected MASLD).

These reactions contrasted with those who drank alcohol problematically or those who recognised they might be at an elevated risk of liver damage and expected an abnormal result. While some individuals in this group expressed initial concern, for most, the immediacy of the result helped mitigate any worries or concerns that might arise over long wait periods for test results. Additionally, how the screening team communicated the results also helped minimise any initial concerns related to their results.

The feedback was you know pretty much immediate. Like seeing it and that’s what you want to hear. There’s nothing worse than waiting around at home and someone says, there’s probably definitely something bad going on inside you. Yeah, it was a swift response. Everything explained…. It’s nothing fatal. Calm down. Everything I wanted to hear. In a way I wanted to hear it (male, Irish, abnormal result, suspected ARLD).

Service providers also acknowledged the service’s effectiveness in engaging individuals facing multiple health disadvantages and connecting them with various forms of healthcare and support. This was particularly evident among those required to attend follow-up appointments, where nurses’ follow-up phone calls prompted discussions about additional forms of support:

I phone them up and we’re on the phone for a five, six min conversation. Just talking about what they’ve done, or some of them have talked to me about their housing situation, like, they want to get out of this house. Yeah, so it’s not just all about the appointment they’ve got. It then broadens to other parts of their lifestyle (pathway navigator, female).

#### Service optimisation

Many comments highlighted service components that were working well (eg, community engagement through outreach and posters), and therefore, minimal immediate optimisations were agreed on.

However, the comments and suggestions from this study helped inform proposed optimisations to be considered for the wider implementation of the service. These included suggestions relating to the importance of word-of-mouth and social media promotion; unclear or insufficient information following a result; time between screening and follow-up appointments; a lack of privacy in some screening settings and options for contacting the service.

The optimisations proposed for the future included providing information to service users that can be shared with friends and family (to enhance word-of-mouth promotion of the service), offering information about the service and results via videos on an NHS website (to address barriers to understanding the information provided at scans); considering social media as a tool to promote the service (to increase the visibility of promotional materials); being mindful of issues of privacy and confidentiality when delivering screening in group or ‘open’ settings (to address concerns about patient comfort) and providing contact details for service users to ask questions (to expand opportunities for further information or follow-up support).

Further details of the proposed and agreed changes, as well as their priority rating, are provided in the table of changes (online supplemental file 3).

## Discussion

Using a rapid qualitative approach, this service evaluation explored the perceptions of service users and providers towards ‘Alright My Liver?’, a pilot service offering rapid point-of-care assessment by TE for liver disease in people at risk.

Among service users, the ‘Alright My Liver?’ pilot service was considered an appropriate and accessible approach to the early detection of liver disease, particularly for service users facing barriers to traditional forms of healthcare assessment, including those experiencing homelessness and people using drugs or alcohol. Consistent with previous work, highlighting that the rapid and tangible feedback from TE is valued by service users,^13^ the immediacy of TE results was similarly appreciated here and helped overcome the barrier of prolonged wait times for results associated with testing through primary care. Participants also consistently cited the accessibility and convenience of co-located liver screening at existing services and community events as a motivation for engagement. This extends existing evidence of the acceptability of TE as a liver screening measure in primary care settings^33^ and drug and alcohol services.^20^

Most participants also reported positive experiences of the service, including its convenience, provision of health-related information and engagement methods. Nevertheless, several optimisations were identified to improve the accessibility and effectiveness of the intervention, which can inform the future delivery of liver health programmes among underserved and at-risk populations.

A key finding was that some participants were prompted to undergo screening due to current or previous heavy alcohol use, either to reassure themselves or to explore the extent of possible liver damage. While this reinforces findings regarding the acceptability of TE among those at risk of alcohol-related liver disease in community settings,^34^ a notable finding was that some participants with suspected MetALD and MASLD sought screening only after experiencing symptoms, receiving signposting, or due to the convenience of the service rather than due to self-perceived risk. This suggests a potential gap in liver health literacy among groups at risk of liver disease of non-alcohol aetiology, particularly regarding the conditions, its symptoms and long-term health conditions, emphasising the need for targeted educational initiatives to enhance awareness of other risk factors for liver disease and reduce wider barriers to healthcare seeking among this population.^35 36^

The majority of participants with normal results indicated that they would consider making lifestyle changes following their scan. This challenges anecdotal concern that a normal result might provide false reassurance around risky behaviours. This also indicates that ‘Alright My Liver?’ was successful in enhancing people’s understanding of liver health and prompting future healthier behaviours which may have a wide-reaching impact. Indeed, although previous research has indicated poor liver health knowledge among at-risk groups, including people who inject drugs^20^ and people experiencing homelessness,^37^ there is evidence that providing tailored information (eg, TE scorecards) can ‘personalise’ results, thereby improving the capacity to understand and apply new liver knowledge.^20^ Indeed, as part of ‘Alright My Liver?’, the provision of individualised advice, including brief alcohol reduction interventions and cessation advice, was particularly valued among participants in this respect, although there was some evidence that those with abnormal results may require further support in periods between follow-up appointments to alleviate short-term anxiety and distress.^33^

### Strengths and limitations

Recruitment through a range of service delivery settings ensured a diverse sample of service users reflecting the target users of the pilot service, including a range of ethnicities and risk factors for liver disease. In particular, a majority of service users interviewed (n=12) had a history of problematic substance use, which is the leading cause of liver-related mortality, and therefore, a key population that the pilot seeks to engage.

The majority of service users interviewed had a normal result from their screening (n=8), which is likely to have an impact on their perception of the service. However, views and experiences did not differ between those with normal compared with abnormal results.

The sample encompassed people at risk of all the major causes of steatotic liver disease but with a majority at risk of ARLD. This was intentional, as the pilot service itself has a focus on people at risk of ARLD, given that it dominates liver-related mortality in the UK and that there are more established pathways to diagnose MASLD cirrhosis.^38 39^ However, this led to an under-representation of individuals with MASLD risk factors, which may also be attributed to possible lower liver-health literacy within this group (as detailed in the discussion).

Despite attempts made, it was not possible to interview any potential service users who declined the intervention. While this could have offered unique insights into barriers to engagement, it is inevitable that people who do not engage with the intervention will be less likely to engage with research relating to the intervention. Nevertheless, qualitative findings related to non-engagement (eg, fear of test results) offer valuable insights into why people may decline screening and can help improve engagement in future events.

Data are not yet available relating to longer-term outcomes for the group of patients found to have liver disease, which would contribute to evidence that the intervention influences behaviour. Similarly, cost-effectiveness data are not yet available to support the intervention.

The findings from this study may be of limited generalisability globally, given the public healthcare system setting.

### Implications for future research and practice

The service evaluation identified that much of the participation in liver disease screening was due to its convenience as a rapid service offered opportunistically, which highlights the importance of community outreach in reaching the population at risk of liver disease and the utility of co-locating screening with existing, trusted ‘walk-in’ services. To address the diverse needs of at-risk populations, further collaborative efforts between healthcare providers, community organisations and policymakers should therefore be pursued when delivering future screening programmes.

Participants with normal results indicated that the screening intervention delivered by the nurse specialist was a catalyst for behaviour change, with many reporting tangible changes to their lifestyle as a result. This indicates that liver screening in at-risk populations may have a role in disease prevention as well as early detection. Future evaluations are required to assess the extent to which liver screening services lead to behaviour change and clinical benefit.

While regular follow-up and HCC surveillance for people with cirrhosis are well-established as necessary and attendance is known to be suboptimal,^39 40^ there is little published work around the facilitators and barriers to this.^41^ This service evaluation has identified the value of interpersonal engagement as well as the provision of taxis to appointments as key drivers in improving engagement with services. Ongoing communication and advice following the initial referral to the hepatology clinic may help address concerns about the time to follow-up and support engagement in behaviour change. These findings are likely applicable across most outpatient services.

## Conclusion

Our findings demonstrate that among service users and providers, the screening aspect of ‘*Alright My Liver?’* was considered an appropriate and accessible early detection screening service for liver disease in high-risk groups. Further work establishing efficacy, clinical benefit and cost-effectiveness is essential to establish the utility of this pilot service, which is likely to have implications for the commissioning of similar services.

## supplementary material

10.1136/bmjgast-2024-001560 online supplemental file 1

10.1136/bmjgast-2024-001560 online supplemental file 2

10.1136/bmjgast-2024-001560 online supplemental file 3

## Data Availability

All data relevant to the study are included in the article or uploaded as supplementary information.
