# Supplementary material for: Qualitative service evaluation of a multimodal pilot service for early detection of liver disease in high-risk groups: ‘Alright My Liver?’
Source: BMJ Open Gastroenterol. 2024 Nov 12;11(1):e001560. doi: 10.1136/bmjgast-2024-001560 (PMC11575350; doi:10.1136/bmjgast-2024-001560)
Supplement: online supplemental file 1 [file bmjgast-11-1-s001.pdf]

Service user interview topic guide

Below is the abbreviated topic guide that university researchers used to conduct the interviews with service users.

Characteristics

1. How old are you?
2. What is your ethnicity?
3. What is your highest educational qualification?
4. Which GP practice are you registered with?
5. Do you live with anyone? What is your housing status?
6. What is your first language?
7. Are you currently working?
8. Do you have a diagnosed health issue for which you are receiving treatment (either primary or secondary care services)? If so, what is the issue?
9. Do you have an undiagnosed health issue?
10. Have you used liver services in the past?
11. Are you currently using liver services?
12. What is your experience of liver services?
13. Are you using alcohol?
14. Are you using drugs?
15. Do you have any other concerns about your health? Including weight status?

Recruitment and willingness to participate

16. What are your reasons for agreeing to participate in the service and continuing to do so after the service was explained?
17. What do you see as the purpose of the service?
18. What do you see as the role of [names of service providers]?
19. What did you/do you hope to get out of the service?
20. What did you think about how you were approached?
21. Could the way you were invited to participate be improved at all? If so, how?

PROMPTS
Referral from GP, etc.
Concern about liver
Concern about impact of alcohol consumption

Experience of service

22. Where did you use the service? What do you think about the service being offered [in XXX]?
23. How long have you been engaged with the service for (screening vs longer term surveillance)?
24. Have you finished your engagement with the service now or have you been invited to a follow-up appointment?
    - If so, can you tell me how your involvement with the service came to an end?
25. Can you talk me through what happens in the service?
    - What do/did you discuss with service providers?
26. What did you think about your session with service providers?
    - What things worked well/did you like?
    - What one thing did you find to be most helpful and why?

- What things worked less well/ you liked less?
- What things helped/benefitted you? How?
- What things did not help/benefit you? How?
- Is there anything you would like to change about the service and the way it ran?
- How acceptable or appropriate was the service?
- Would you recommend this service to family/friends?
- What did you think of how the service provider delivered the service? What did you like/dislike about their approach? (e.g. views on formality of the service)

PROMPT for views on each component of the service: Introduction / explanation of test, offer of BBV, liver scan procedure, verbal and written advice received, result of scan, referral to hospital clinic

27. Do you feel more informed about liver health because of the service?
28. If referred to clinic: Can you talk me through what happens when referred to hospital? (e.g. ultrasound and blood tests to confirm results, then clinic appointment with Dr)
29. What do/did you discuss with service providers? Confirmation of results? False positives?
30. Were you contacted before the appointment? (phone call, offer of transport)
31. What things worked well/did you like?
32. What one thing did you find to be most helpful and why?
33. What things worked less well/ you liked less?

PROMPTS: appointment waiting times

34. What did you think about your session with service providers?
35. What things helped/benefitted you? How?
36. What things did not help/benefit you? How?
37. Is there anything you would like to change about the service and the way it ran?
38. How acceptable or appropriate was the service?
39. Would you recommend this service to family/friends?
40. What did you think of how the service provider delivered the service? What did you like/dislike about their approach? (e.g. views on formality of the service)

Experiences of service impact

41. What has changed since the service?
42. Has the way you view your health changed? If so, how?
43. Have you/do you plan to make any changes following the screening? For example, do you think the way you manage your health [will] change[d] at all? If so, how?
44. Has your feeling of wellbeing changed at all? If so, how?
45. Has your quality of life (e.g. ability to work, socialise, do things they love) changed at all? If so, how?

Barriers and facilitators to service

46. Were you given any advice? If yes, what advice?
47. Did you receive a leaflet covering information about the service, the liver, results, looking after your liver in relation to alcohol consumption, non-alcohol fatty liver disease and, viral hepatitis?

48. If yes, what did you think about this leaflet? Likes/dislikes?
49. If no, what do you think about this?
50. What advice would you find helpful? (e.g. liver health, drink, weight related advice)
51. What things [do you think will help] / helped use the strategies developed with service providers?
52. What things [do you think will help] / made using the strategies developed with service providers difficult?
53. Are there any challenges in attending the sessions / being part of the service screening and/or follow-up appointments?

PROMPTS
Opinion/relationship with service providers
Difficulties attending appointments

Factors to consider for future service development

54. What are the key ingredients needed for this service to run successfully in the future?
55. What things would support the service being rolled out more widely?
56. What things would need to be addressed/improved for service to be rolled out more widely?
57. Where else do you think it would be good to offer this service?
58. What do you think would encourage other people to use the service? E.g. incentives?

Continued issues or challenges

59. Can you tell me a bit about any continued issues or challenges you face in relation to your liver health or health in general?
60. Has anything changed since your involvement with the service ended?
