# Supplementary material for: Qualitative service evaluation of a multimodal pilot service for early detection of liver disease in high-risk groups: ‘Alright My Liver?’
Source: BMJ Open Gastroenterol. 2024 Nov 12;11(1):e001560. doi: 10.1136/bmjgast-2024-001560 (PMC11575350; doi:10.1136/bmjgast-2024-001560)
Supplement: online supplemental file 2 [file bmjgast-11-1-s002.pdf]

Service provider interview topic guide

Below is the abbreviated topic guide that researchers used to conduct interviews with service providers.

1. Overview of service and role
Describe the service
Describe role within the service

2. Experience of service and role
Things that worked well
Things that worked less well
Things that could be improved
Adverse events identified
Things that helped/benefitted patients
Things that helped the patients less/benefit patients less well
Acceptability/ appropriateness of the service among staff and patients
Things liked among staff and patients
Things not liked among staff and patients

PROMPTS
Identifying eligible patients
Partnership working/communication between service(s)/ providers
Data sharing between outreach events and hepatology clinics

4. Barriers and facilitators to service
Patient facing model
Facilitators of the service
Barriers to the service

5. Factors to consider for future service development
Things that would support the service being rolled out more widely
Things that would need to be addressed/improved for the service to be rolled out
