# Supplementary material for: Qualitative service evaluation of a multimodal pilot service for early detection of liver disease in high-risk groups: ‘Alright My Liver?’
Source: BMJ Open Gastroenterol. 2024 Nov 12;11(1):e001560. doi: 10.1136/bmjgast-2024-001560 (PMC11575350; doi:10.1136/bmjgast-2024-001560)
Supplement: online supplemental file 3 [file bmjgast-11-1-s003.pdf]

Table of changes

Coding framework		
Code	Stands for	Means
IMP	Important for behaviour change	This is an important change that is likely to impact behaviour change or a precursor to behaviour change (e.g. acceptability, feasibility, persuasiveness, motivation, engagement), and/or is in line with the Logic Model, and/or is in line with the Guiding Principles For example, participants appear unconvinced by an aspect of the intervention, so you decide to add motivational examples.
EAS	Easy and uncontroversial	An easy and feasible change that doesn't involve any major design changes. For example, a participant was unsure of a technical term, so you add a definition.
REP	Repeatedly	This was said repeatedly, by more than one participant.
EXP	Experience	This is supported by experience. Please specify what kind of experience, for example:<br>1. PPIs agree this would be an appropriate change.<br>2. Other stakeholders (e.g. practitioners, providers, topic specialists) agree that this would be an appropriate change.<br>3. Literature: This is supported by evidence in the literature.
NCON	Does not contradict	This does not contradict experience (e.g. evidence), or the Logic Model, or the Guiding Principles
NC	Not changed	It was decided not to make this change. Please explain why (e.g. it would not be feasible; or only one person said this).
MoSCoW	Must have, Should have, Could have, Would have	Optimisation prioritisation rating as agreed by service provider team following review of results
I	Interviewer	-
P*	Participant	-
SP*	Service provider	-

A pilot service offering early detection of liver disease in high-risk groups: "Alright My Liver?"						
Intervention element	Negative Comments	Positive Comments	Possible Change	Reason for	Agreed change / NC	MoScoW

				chan ge		
**Outreach: Trusting relationships/rappor t building**		P1: She is I can tell a good manager she is a good talker at the same time. She knows how to approach people and you know influence them and all that, that's how she got me the first time, everything was very well and bingo finally I'm like all my people you have to do this thing you and I'm going to bring you all, I'm gonna bring you all my people.	No change	NC – Work ing well	NC	M
**Outreach: Engaging with service providers**		SP6: Yes, we have a relatively big clinic room, they set up down there. And we shuffle people towards them when they come in. So that's been quite nice because I think lots of our clients sort of see it as part of the service as well rather than an external agency. Which I think also helps bring a bit of trust in as well which works quite well.	No Change	NC – Work ing well	NC	
**Outreach: social media**		P1: That's what you've got to do and nowadays people are also on social media I don't know if social media?	Social media strategy to increase awareness of service	EAS	Discuss social media strategy with team, being mindful of barriers to using social media in NHS contexts.<br><br>Explore costs of advertising on	C

					social media.	
**Outreach: Music to draw people in**		P1: I think sometimes people like music also, people can upshot people and if there's some music they will come and see what's going on.	Play music to attract attention and draw people in	EAS	No change – only one participant and some people may dislike this.	W
**Outreach: WhatsApp**		P4: Mostly through WhatsApp, especially this group because they post a lot of things. So I mostly check in the morning, and if I see something I'm interested in I just go and visit them. And this one, when I saw it I called my sister and I said, 'Oh, you have a liver problem, why don't you go and check it, they have this effect in the [6:29 inaudible] team this weekend, and she checked it, yeah.	Invite collaborating service providers share information on any WhatsApp channels.	EAS	Invite collaborating service providers share information on any WhatsApp channels.	S
**Process: Information leaflet**	I: Is there anything you think you would change about how the service was being run or how the actual sessions was being run when you were inside the room with Sally?<br><br>P5: Possibly just something that you could take away, that probably gives you a bit more information because, to be honest, when I was doing the scan, it's the first time I was even aware where the liver was located. So, maybe	P12: I mean some people like reading. I mean I'm not much of a reader. Like obviously I do read, but generally I get given leaflets. I went to AA meeting; I got a load of leaflets and they've been sat up in my room for like a month and a half now.<br><br>I: Really, yeah, yeah.	Provide information in alternative formats	REP, NCON	Create information videos for website	S

	just some information. She did tell you about aftercare, maybe if you put on a bit of weight, you know, how that would affect the liver, stop the liver from working. So, there was a bit of information but if you could get that to take away and just a bit more about the liver for people who don't understand that.<br><br>P13: Yes I don't know whether I've got to give up drinking forever or am I doing it wrong like when I go on holiday and having a drink and that, am I harming myself.	P12: Do you know what I mean. I just don't get around to reading them.<br><br>I: Do you think there's a better way that people can deliver it.<br><br>P12: Sit down and chat to us about it. If they've got the time. Maybe spend another 10 minutes. You know, if somebody's got a problem, sit down and chat to them for 10 minutes. That'd be good, but obviously they're trying to get other people done, so it's not always possible				
**Process: Phone line to allow people to call and ask questions about the service before attending**	P5: I know you're going to the learning centre next and I know a lot of people go to the learning centre so leaving information there that people can access but also there is a number there that they can call and speak to someone about it if they need to because I do think it's a very important service and it would get a very big uptake, I do believe.		Provide phone line to call up and ask questions	NC – only one participant suggested this and it is too costly for the	Provide email address for questions about the service.<br><br>Mobile number is provided for those with reactive results to ask questions.	W

				current service		
**Process: Information given at the start: It's not going to hurt**	P6: I think it would be good because it's painless, you don't feel anything. All you feel is a little knock and it's over in two minutes. So, you could give a lot more people a sense of relief.<br><br>I: Is that quite an important thing to say, the fact that it's not going to hurt?<br><br>P6: Yes, going forward, yes, go where you can.		Inform patient before scan that it does not hurt	EAS	Include information that it will not hurt before conducting the scan.	S
**Outreach: Group setting**	I: Is there anything you didn't like about us offering it here?<br><br>P7: I don't know. Not really. Maybe a little bit of peer pressure, but not that much.<br><br>I: OK, so is that from the other people staying here or from the staff?<br><br>P7: Not the staff. It's more like the other people.<br><br>I: So, the fact that you were all asked would you like to do it in front of each other?<br><br>P7: Yes, it's just one of those things like, no-one wants to be the person to say yes first or no first.		Be conscious of issues of privacy and confidentiality.<br><br>Maintain a balance between privacy and accessibility for at-risk groups.	EXP	Ensuring the patient is comfortable with the setting and has privacy and confidentially within the setting.<br><br>Maintain a balance between the need for privacy and accessible settings for at-risk groups.	M

	I: How do you think that could be done differently?<br><br>P7: I'm not sure how you would do it differently to be honest.<br><br>I: Did you feel that once you did go into the room with ((Sally)) you could say no when you were on your own, did she check again?<br><br>P7: Yeah, she said you can stop this at any time, yeah.<br><br>---<br><br>P12: Like some people just don't want other people knowing their stuff and it seems quite open. It seems a bit open in here. People are walking through. There's no privacy in there.					
**Outreach: Posters**		P4: The poster is the quickest.<br><br>---<br><br>P3: Some foreign people say like in my community they don't understand English, if you talk to them they don't understand what you're saying. When you've found somebody who can speak to them language then it's easier and basically around here there's like one Mosque over there and another Mosque down here then you can put letter in the door as	No Change	NC – Working well	NC	M

		well to ask them permission, the very good people then you can put a letter there and tell them what time and when it's gonna be a van and checking and stuff then that's definitely is helping. Or any shop around here like different communities so Somalian, Jamaican, Caribbean, Asian all the shops you can put a sign there or letter then people are going to read it.				
**Outreach: Community members doing outreach**		P5: Yes, definitely and in saying that I'm thinking I might have to put myself forward because I'm very known in the community in [place name]. I know a lot of the people there and I could probably outreach to certain people. So, having somebody from the community who people will open to, I think.	Encourage inviting friends and family<br><br>Develop materials to be shared with friends and family	REP, IMP	Develop materials to be shared with friends and family	C
**Outreach: Word of mouth**		I: So you've brought [name].<br>P1: Yeah I've brought [name].<br>I: You've brought him to come down.<br>P1: And even [service provider location] I've brought a lot of people for scan.<br><br>I: Oh really.<br>P1: Yeah.	Encourage inviting friends and family<br><br>Develop materials to be shared with friends and family	REP, IMP	Develop materials to be shared with friends and family	C

		I: Oh wow.<br>P1: More than 20 people.<br><br>―――<br><br>P2: Yeah, yeah that is good. Everybody at the café, restaurant café I tell everybody you have a scan on my liver.				
**Process: Unclear/or insufficient information following result**	I: And with the Alrightmyliver service have you finished seeing them now or are you still having appointments or where are you with it?<br><br>P10: Um, I haven't, I haven't heard back in a while, a little while but I think they did send me a message they were going to – it was depending. Obviously, he's busy. I understand -<br><br>I: Okay.<br><br>P10: - but yeah and the other thing I'm all – I don't think I'm all done.<br><br>I: Okay. So you think they're going to arrange another appointment.<br><br>P10: Yeah, I think so.<br><br>―――		Provide further information about reactive results in the moment and/or with a follow-up phone call.	IMP	Provide additional information in video format	S

	P12: But they didn't really say a lot about it. They said that it was sort of pre-scarring level and that it was quite high and that I'm going to go to the hospital and get a – speak to a doctor or get another scan done<br><br>P11: They didn't say too much. They just said, oh we can see something on the screen, on the scan. We need to see you up at the ((BRI)) and that was it. It was all very – I always take everything in my stride. I must admit I'm quite laid back. So, I thought, be bold. Go for the appointment. See where we go from there, yeah.					
**Process: Telephone calls and rapport building**		SP5: It has, yeah. The majority of our patients do lead chaotic lifestyles. So, it is difficult to keep them on track. We're going into our second, almost third appointments now. Fortunately, the patients have continued, but it has been a lot of phone calls, or emails to the support workers. The ones that we have, we, sort of, identified at the beginning, like when I first started, I built up a good relationship with them, they know when I'm phoning them, they know it's me on the phone now, and almost having a bit of a chit-chat about what's going on and stuff	No change	NC – Working well	NC	M

**Process: Taxi provision**		I: Yes okay, but was that helpful for you having the taxi?<br><br>P13: Yes it was yes.<br><br>I: Yes, do you think you would have gone if you didn't have -<br><br>P13: No I don't think I probably would have.<br><br>I: Really, yes, yes, and that's because of the charge you think?<br><br>P13: Yes the charge, I mean I'd have to park up on top of some ((St Michaels Hill))[place name] [inaudible 20:30] not very good but it made it all very convenient for me to go.	No Change	NC – Working well	NC	S
**Process: Comprehension of result**			Create information videos<br><br>Provide mobile number for those with a reactive result<br><br>Provide email address for any questions.	IMP	Create information videos<br><br>Provide mobile number for those with a reactive result<br><br>Provide email address for any questions.	M
